# Cytosolic *Nudix Hydrolase 1* Is Involved in Geranyl *β*-Primeveroside Production in Tea

**DOI:** 10.3389/fpls.2022.833682

**Published:** 2022-05-11

**Authors:** Hanchen Zhou, Shijie Wang, Hao-Fen Xie, Guofeng Liu, Lubobi Ferdinand Shamala, Jingyi Pang, Zhengzhu Zhang, Tie-Jun Ling, Shu Wei

**Affiliations:** ^1^State Key Laboratory of Tea Plant Biology and Utilization, Anhui Agricultural University, Hefei, China; ^2^Tea Research Institute, Anhui Academy of Agricultural Sciences, Huangshan, China; ^3^Henan Provincial Key Laboratory of Tea Plant Biology, Xinyang Normal University, Xinyang, China

**Keywords:** *Camellia sinensis*, geraniol biosynthesis, geranyl *β*-primeveroside, Nudix hydrolase 1, tea aroma

## Abstract

Geraniol is a potent tea odorant and exists mainly as geranyl glycoside in *Camellia sinensis*. Understanding the mechanisms of geraniol biosynthesis at molecular levels in tea plants is of great importance for practical improvement of tea aroma. In this study, geraniol and its glycosides from tea plants were examined using liquid chromatography coupled with mass spectrometry. Two candidate geraniol synthase (*GES*) genes (*CsTPS*) and two Nudix hydrolase genes (*CsNUDX1-cyto* and *CsNUDX1-chlo*) from the tea genome were functionally investigated through gene transcription manipulation and gene chemical product analyses. Our data showed that in tea leaves, levels of geranyl *β*-primeveroside were dramatically higher than those of geranyl *β*-glucoside, while free geraniol was undetectable in this study. A tempo-spatial variation of geranyl *β*-primeveroside abundance in tea plants existed, with high levels in young and green tissues and low levels in mature or non-green tissues. Cytosolic CsNUDX1-cyto showed higher hydrolysis activity of geranyl-pyrophosphate to geranyl-monophosphate (GP) *in vitro* than did chloroplastidial CsNUDX1-chlo. A transgenic study revealed that expression of *CsNUDX1-cyto* resulted in significantly more geranyl *β*-primeveroside in transgenic *Nicotiana benthamiana* compared with non-transgenic wild-type, whereas expression of *CsNUDX1-chlo* had no effect. An antisense oligo-deoxynucleotide study confirmed that suppression of *CsNUDX1-cyto* transcription in tea shoots led to a significant decrease in geranyl *β*-primeveroside abundance. Additionally, *CsNUDX1-cyto* transcript levels and geranyl *β*-primeveroside abundances shared the same tempo-spatial patterns in different organs in the tea cultivar “Shucha Zao,” indicating that *CsNUDX1-cyto* is important for geranyl *β*-primeveroside formation in tea plants. Results also suggested that neither of the two candidate GES genes in tea plants did not function as GES in transgenic *N. benthamiana*. All our data indicated that *CsNUDX1-cyto* is involved in geranyl *β*-primeveroside production in tea plants. Our speculation about possible conversion from the chemical product of CsNUDX1-cyto to geranyl *β*-primeveroside in plants was also discussed.

## Introduction

Geraniol is an acyclic monoterpene alcohol produced by many plant species, including rose (Baydar and Baydar, 2005) and ginger grass (*Cymbopogon martinii*; Dubey and Luthra, 2001). It is a signaling molecule which plays key roles in plant-environment interactions, such as pest repelling (Wei et al., 2004b), antimicrobial activity (Inouye et al., 2001), pollinator attraction (Magnard et al., 2015), and priming cold tolerance in adjacent plants to enhance plant resistance to cold stress (Zhao et al., 2020). Geraniol possesses a pleasant and sweet rose odor with perception threshold values ranging from 4 to 75 ppb (parts per billion; Burdock, 2001), and also exhibits various biochemical and pharmacological properties. It has been widely used for household, cosmetic and pharmaceutical products (Chen and Viljoen, 2010). Moreover, like some other plant aroma compounds, geraniol also affects human food acquisition, and aroma is a consumer preference determinant of tea products (*Camellia sinensis*). Geraniol is one of the most abundant volatiles in tea (Ho et al., 2015), and imparts its unique floral scent to different tea varieties (Wang et al., 1993; Ubota, 2002; Gui et al., 2015; Han et al., 2016; Kang et al., 2019).

Unlike the floral scent produced from intact flowers, vegetative scents are often generated after vegetative tissue damage (Dong et al., 2016). Fresh and intact tea leaves emit very few volatile odorants and possess no smell, but they may contain a substantial amount of glycosidically conjugated geraniol, which is thought to be involved in detoxification of lipophilic and membrane-toxic terpenols (Stahl-Biskup et al., 1993) and regulation of signal transduction in plants (Bock, 2015). The conjugated volatile terpenoids can be released upon tissue disruption (Takeo, 1981) and contribute to tea aroma through the processing procedure (Ho et al., 2015). In many cultivars of *Camellia sinensis*, the main glycoside of geraniol is geranyl *β*-primeveroside (Wang et al., 2000). It has been recently suggested that formation of geranyl *β*-primeveroside in tea shoots results from sequential glycosylation reactions driven by uridine diphosphate glycosyltransferases CsGT1 and CsGT2 (Ohgami et al., 2015). Biosynthesis of geraniol or its precursor is considered to be the starting point for geranyl *β*-primeveroside formation and accumulation in tea plants. However, no geraniol synthase (*GES*) genes have been identified in tea plants, while many *GES* genes have been found in other plant species (Degenhardt et al., 2009). Interestingly, it was once suggested that a leafhopper geraniol synthase was likely contributing to tea geraniol production and the characteristic aroma of “Oriental Beauty” tea (Zhou et al., 2019).

**Figure 1:**
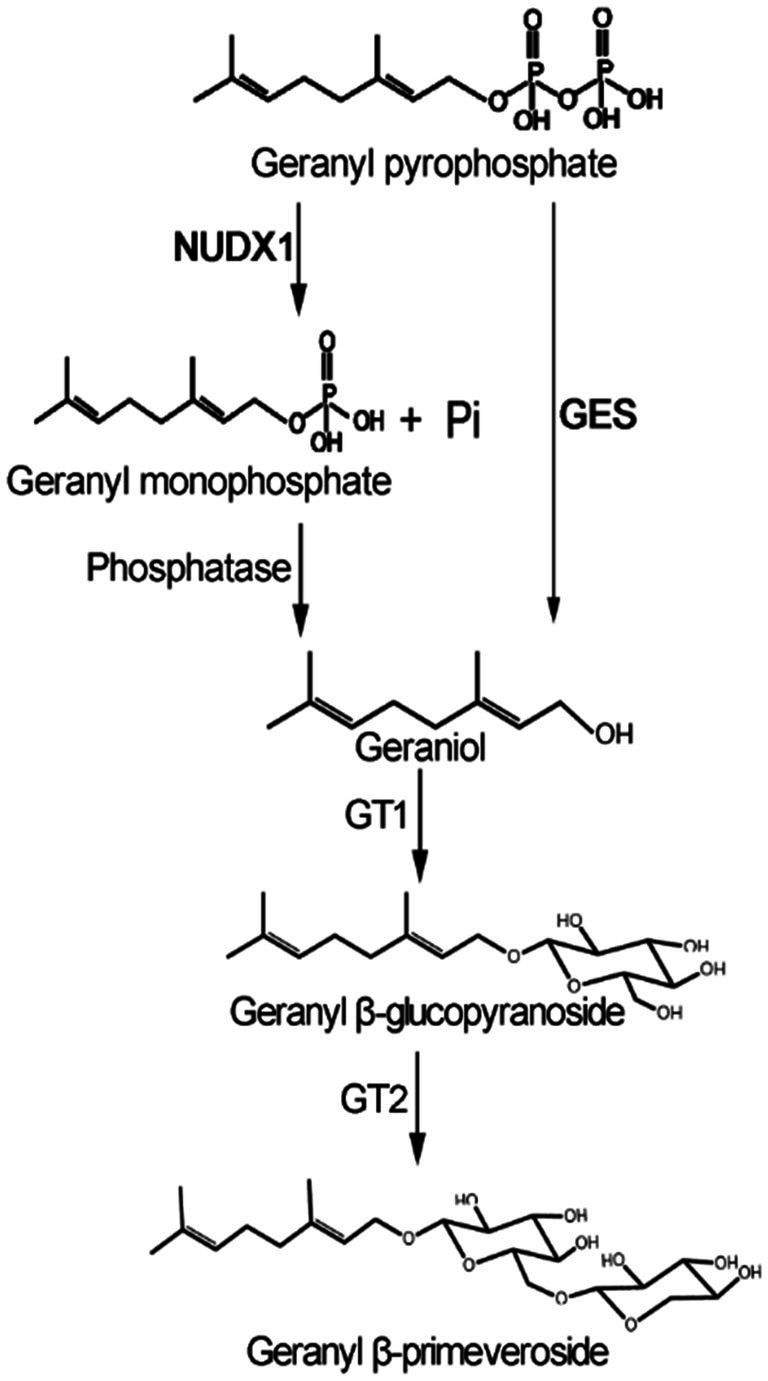
Possible pathways for geraniol and its glycoside production in tea plants. GES, geraniol synthase; NUDX1, Nudix hydrolase1; GT1, UDP-glycosyltransferases; GT2, UDP-xylosyltransferase.

In plants, geraniol and other monoterpenes are usually produced by corresponding terpene synthases (TPS) through the terpenoid biosynthesis pathway. This knowledge has been applied towards enhancement of crop product aroma. For example, sweet basil geraniol synthase (*ObGES*; Iijima et al., 2004) has been successfully employed to enrich geraniol and its derivatives (Davidovich-rikanati et al., 2007) in tomato fruit for flavor improvement. Recently, a noncanonical pathway for geraniol production has been revealed in a rose hybrid, in which a cytosolic Nudix hydrolase (RhNUDX1) was found to catalyze phosphohydrolysis of geranyl pyrophosphate (GPP) into geranyl monophosphate (GP), followed by dephosphorylation with an endogenous phosphatase to produce geraniol (Magnard et al., 2015). It is of interest to find out whether the crucial genes in the canonical and/or noncanonical pathways shown in Figure 1 are functional in tea plants for geraniol and geranyl *β*-primeveroside biosynthesis.

In this study, the crucial genes in the putative canonical and noncanonical pathways for biosynthesis of geraniol and its *β*-primeveroside in tea plants were investigated for the ultimate purpose to improve geraniol-specific tea aromas. Our data indicated that the noncanonical pathway through *CsNUDX1-cyto* is involved in tea geranyl *β*-primeveroside production. These findings will promote a further elucidation of tea geraniol and its glycoside production at molecular levels, and may have the potential to improve tea geraniol-related aroma through targeted molecular breeding approaches.

## Materials and Methods

### Materials

The following 10 cultivars of *C. sinensis* var. *sinensis* maintained in the Guo-He Tea Germplasm Collection of this university (Hefei, China) were used in this study: “Shucha Zao” (“SCZ”), “Echa NO.1” (“EC1”), “Echa NO.5” (“EC5”), “Fuding Dabai” (“FDDB”), “Mingshan Baihao” (“MSBH”), “Mingxuan 213” (“MX213”), “Wancha 91” (“WC91”), “Zhongcha 108” (“ZC108”), “Zhongcha 102” (“ZC102”), and “Zhenong 117” (“ZN117”). Tea plant samples were all excised from tea cultivars, immediately deep frozen in dry ice and then brought to the lab for storage at −80°C for further use. For transgenic studies, plants of *Nicotiana tabacum* cv. “Yunyan 85” and *Nicotiana benthamiana* were grown in pots containing a peat and vermiculite mixture (1:3, *v*/*v*) and kept in a growth chamber with a 16 h photoperiod of 150 μM m^−2^ s^−1^, at temperatures of 22°C/25°C (night/day) and 60% relative humidity. Plants were regularly fertilized and watered.

For chemical analysis, geranyl *β*-primeveroside and linalyl *β*-primeveroside were synthesized by Na-Fu Biotech (Shanghai, China)^1^ and further verified with nuclear magnetic resonance spectroscopy (Supplementary Table S1). Geranyl *β*-D-glucopyranoside was synthesized by Prof Zhengzhu Zhang’s group at Anhui Agricultural University. Geraniol, citral, linalool, geranyl diphosphate, and geranyl monophosphate were purchased from Sigma (Shanghai, China).

### Volatile Collection From Tea Infusion and Plant Tissues and GC-MS Analysis

For geraniol quantitative analysis of tea infusion, black teas were made according to a local black tea processing protocol (DB34/T1086-2009) using the four widely-grown tea cultivars (“WC91,” “ZC108,” “ZN117,” and “SCZ”) in Qimen county in the Tea Research Institution, Anhui Academy of Agricultural Sciences. Briefly, one or two leaves with one bud were plucked from Qimen, Anhui Province, China and spread indoors for 4–6 h. Subsequently, a hot wind blower was used for withering the tea leaves (for 6 h at 30°C), followed by rolling for 60 min and then fermented for 120 min at 26°C. The tea leaves were further dried and then stored at −40°C for aroma analysis. Head-space abundances of geraniol emitted from tea infusions of different black teas were analyzed together with other volatiles using gas chromatography–mass spectrometry (GC-MS) and were performed in triplicate. The tea infusion volatile collection and analysis were conducted according to Han et al. (2016) with a slight modification. Each tea sample (0.5 g) was placed into a 20 ml vial with 5 ml boiling distilled water and kept for 5 min in a water bath at 70°C. An SPME fiber (65 μM PDMS/DVB Stable Flex, Supelco Inc., Bellefonte, PA, United States) was used to collect the volatiles for 30 min, then 5 μl ethyl decanoate (0.01%) was added to the samples as the internal standard. An Agilent 7697A GC coupled to an Agilent 7890A MS system was used to conduct the volatile analysis. Linalool and geraniol identification and quantification were performed using authentic standards according to a previously published protocol running for 45.0 min (Han et al., 2016). For better resolution of volatiles collected from tea infusions, the GC program was modified to run for 59.6 min with a slow increase in temperature.

Enzymatically released geraniol analysis was conducted according to Wei et al. (2004a) with some minor modifications: 200 mg fresh samples were homogenized in liquid nitrogen and 20 U almond *β*-glucosidase (Aldrich-Sigma, Shanghai, China) was added to the homogenized leaf sample, followed by 30 min incubation at 37°C prior to headspace SPME collection and GC–MS analysis as previously described.

### Database Search for Tea Candidate Genes for Geraniol Biosynthesis

*RhNUDX1* (GenBank accession number JQ820249; Magnard et al., 2015) and functional geraniol synthase genes in sweet basil and another six plant species, were used as query sequences to retrieve their corresponding homologues from the tea genome (Wei et al., 2018; Xia et al., 2019) by BLASTX. Prediction of protein subcellular localization was performed using the online software ChloroP1.1.^2^ Protein sequences of the two tea *NUDX1* genes obtained by BLASTX, *RhNUDX1*, and the Arabidopsis homologue (AT1G68760) were compared using ClustalW2.^3^

### Prokaryotic Expression and Enzymatic Analysis

Coding sequences for the two tea Cs*NUDX1* genes were subcloned into the *Nde* I/*Xho* I sites of pET15b and the functional Arabidopsis homologue AtNUDX1 was used as a positive control (Liu et al., 2018b). The resulting chimeric genes were separately introduced into the *Escherichia coli* BL21 (DE3) Rosetta strain. Prokaryotic protein expression, His-tag removal, protein purification, and *in vitro* enzymatic activity determination were carried out as previously described (Liu et al., 2018b). The reaction products were then analyzed exactly as previously described on a Shimadzu 20 AD UFLC system coupled with a Qtrap 5500 (AB Sciex, United States; Liu et al., 2018b). Five microliters of reaction product from each assay was used for product analysis using authentic standards as controls. The mass spectrometer was operated in the negative ionization mode using multiple reaction monitoring (MRM) for the transitions of *m*/*z* 313.10 to 78.9 and 233.10 to 78.9 (corresponding to GPP and GP, respectively) with the previously optimized parameters (Liu et al., 2018b).

### Subcellular Localization Analysis

The two tea CsNUDIX1s were fused at their C-termini with green fluorescent protein mGFP5 (GenBank U87973.1) through recombinant polymerase chain reaction (PCR) using gene specific primers (Supplementary Table S3) and then subcloned into pBP121 between the *Xba* I and *Sac* I sites. The plant expression vectors containing the GFP fusion constructs were then separately introduced into *Agrobacterium tumefaciens* strain GV3101, and these were used for transient expression in *N. benthamiana* as conducted earlier (Liu et al., 2018a). GFP fluorescent signal was observed 3 days after the infiltration using an Olympus FV1000 confocal laser scanning microscope (Olympus Corporation, Beijing, China) with excitation and emission wavelengths of 484 and 507 nm, respectively.

### Functional Characterization in Transgenic Tobacco Plants

Each of the coding sequences of the four candidate genes was subcloned into the *Xba* I and blunted *Sal I* sites of pCAMBIA2300 downstream of the Cauliflower mosaic virus 35S (CaMV 35S) promoter (Cambia, Canberra, Australia) and introduced into *A. tumefaciens* (GV3101) for *N*. *benthamiana* leaf transient expression according to Sparkes et al. (2006). The geraniol synthase gene (*ObGES*; AAR11765.1) from sweet basil (*Ocimum basilicum* L. cv. Sweet Dani; Iijima et al., 2004) was used as a positive control, and the non-transgenic wild type was used as a negative control. Transformed and control leaves were then excised for chemical analysis.

### Suppression of *CsNUDX1* Expression in Tea Plants

To suppress plant endogenous target gene expression, the approach using antisense oligonucleotides (asODN) was employed, and the 10 top candidate asODNs were obtained using tea NUDX1 sequences as inputs into Soligo software (Ding and Lawrence, 2003; Dinç et al., 2011). These asODNs-CsNUDX1-cyto were synthesized by Invitrogen (Shanghai, China), and each asODN was prepared at 20 μM. “SCZ,” an elite cultivar of *Camellia sinensis* var. *sinensis* with publicized genome sequence data and abundant geraniol and its glycoside, was selected for *CsNUDX1* suppression study. Field-grown tender tea shoots, each with three unfolded leaves, were excised and inserted into Eppendorf tubes containing 1 ml of 20 μM AsODN-CsNUDX1-cyto. These tea shoots were maintained in a growth chamber in which temperatures was set at 22°C/25°C (night/day), with a 16 h photoperiod at 60 μM m^−2^ s^−1^ and 60% relative humidity. The asODN solution was added into each tube daily in amounts needed to maintain the solution volume. As a control, a 17-mer random nonsense oligonucleotide 5′-GGCGGCTAACGCTTCGA-3′ was applied according to (Dinç et al., 2011). The shoots were then sampled at different time intervals for gene expression and metabolic profiling. Biological triplicates were performed for each treatment.

### Quantitative Analysis of *CsNUDX1* Transcripts

For gene transcript quantification, total RNA from different organs such as shoot stems and leaves at three different developmental stages of field-grown plants were obtained using the Takara RNAzol kit and then treated with DNaseI (Takara, Dalian, China) to remove contaminating genomic DNA. The cDNA samples were obtained by reverse transcription using the PrimerScript™ RT kit (Takara, Dalian, China). Transcript levels were quantified using quantitative real-time PCR with gene-specific primers (Supplementary Table S3). The reaction system was prepared using TransStart Top Green qPCR Mix Kit (TransGen, Beijing, China) following the manufacturer’s instructions. The reaction volume was 25 μl, and contained 12.5 μl 2× reaction solution, 10 μM primers, 1 μl template and water. The amplification efficiencies of the test were all above 85%. The transcript data were normalized using the internal reference gene 18S rRNA. The gene transcript level was calculated using the 2^−ΔΔ*C*t^ method (Livak and Schmittgen, 2001).

### Chemical Analysis of Plant Extracts

**Figure 2:**
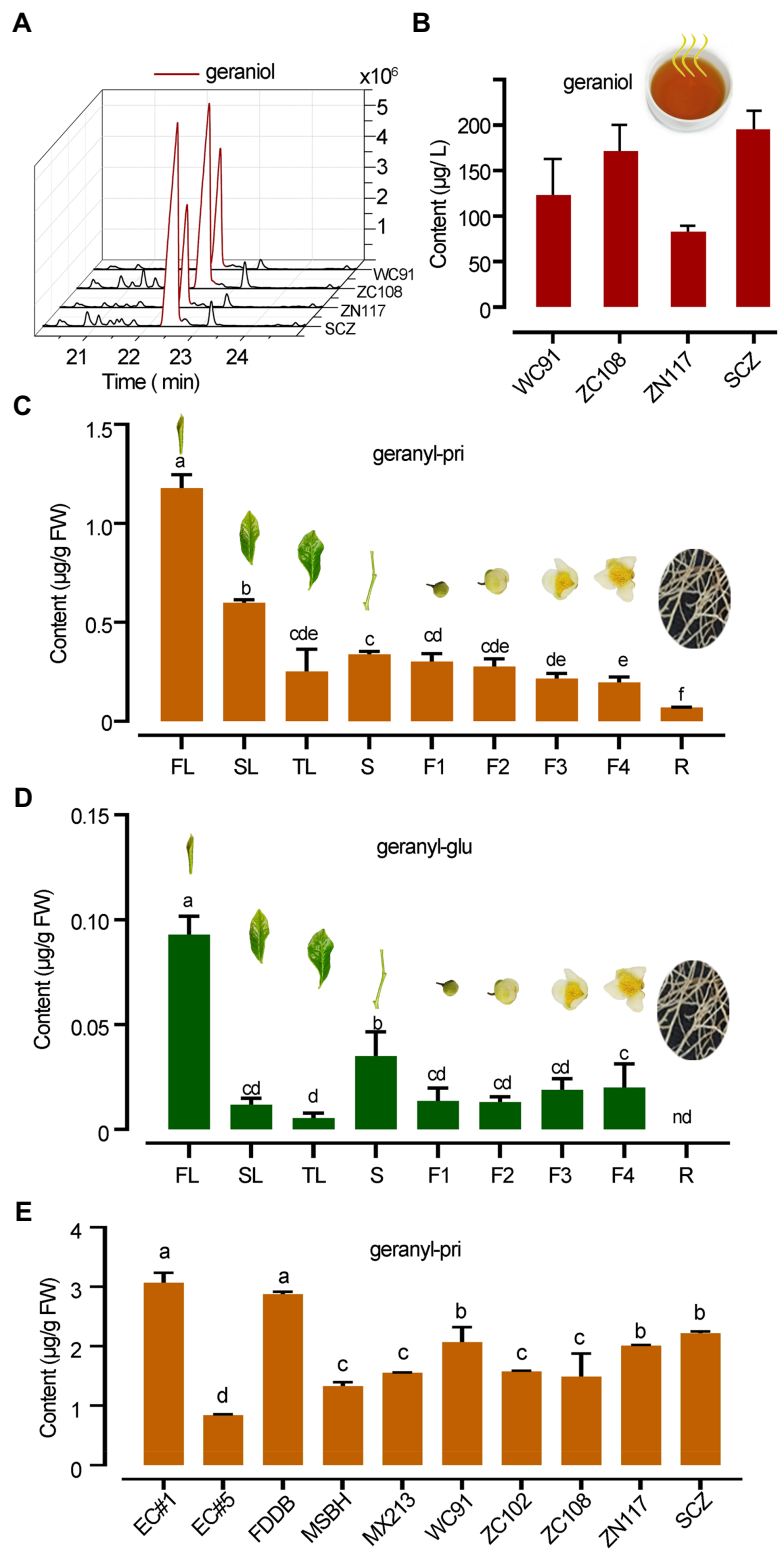
Distinct abundance difference in geraniol and its glycosides in tea. **(A,B)** GC chromatography and quantitative column presentation of geraniol SPME collected in tea infusion headspace of different teas. **(C,D)** The abundances of geranyl primeveroside (geranyl-pri) and geranyl glucoside (geranyl-glu) in different organs of “SCZ” tea plants. FL, SL, and TL, first, second and third leaves; S, tender shoot stem; F1–F4, flowers at different developmental stages; R, tender roots. **(E)** Quantification of geranyl primeveroside in different tea cultivars. Columns with different letters had significant differences in the abundances of the same compound (*p* < 0.05) using the statistical method described in text. EC1, “Echa NO.1”; EC5, “Echa NO.5”; FDDB, “Fuding Dabai”; MSBH, “Mingshan Baihao”; MX213, “Mingxuan 213”; WC91, “Wancha 91”; ZC102, “Zhongcha 102”; ZC108, “Zhongcha 108”; ZN117, “Zhenong 117”; SCZ, “Shucha Zao.”

The elite cultivar “SCZ” was used for tempo-spatial abundance analysis of geranyl glycosides in tea plants. Basipetally the first, second, and third leaves, and tender stems were excised from the currently growing shoots and flowers at four different developmental stages and tender roots were also excised from tea plants as shown in Figure 2. Moreover, variation analysis of geranyl primeveroside abundances among all the above-mentioned 10 cultivars were also performed. For chemical analysis, plant extracts were prepared and analyzed according to a previously published method (Flamini et al., 2014) with minor modifications. Briefly, homogenized plant samples (0.100 g each) were weighed to a precision of four decimal places, and were added to ice-cold methanol that was pre-acidified with 0.1% (*v*/*v*) formic acid based on the ratio of 300 μl/100 mg fresh sample weight. Supernatants were obtained after vortexing, sonication at 36 KHz (KQ-500DE, Kunshan, China) and centrifugation at 4500 RPM for 10 min (D3024, Scilogex, Marietta, GA 30062, United States). The extracts were filtered through 0.22 μm syringe filters (QFC06-SF022N13, Qingfeng, China). Plant extracts were analyzed with ultra-high performance-liquid chromatography-quadrupole time-of-flight mass spectrometry (UHPLC-Q-TOF-MS). Five microliters of the filtered extract were injected into an Agilent UHPLC 1290 Infinity II coupled to an Agilent 6545 Q-TOF-MS with an ESI source (Agilent Technologies, Santa Clara, CA) in the positive or negative ionization mode. Chromatographic separation was performed using a Zorbax reverse-phase column (RRHD SB-C18, 3 × 150 mm, 1.8 μM; Agilent Technologies, Santa Clara, CA) using the gradient elution program and Q-TOF conditions as per Flamini et al. (2014). Compounds were identified based on the comparison of accurate retention times and MS data between samples and authentic standards. The intensity of the identified compound in the samples was represented using the corresponding peak area normalized to the amount of fresh plant tissues.

### Statistical Analysis

All quantitative analyses were performed with at least three biological replicates, and statistical differences among the means were analyzed using ANOVA followed by the Bonferroni test (SPSS statistics 19.0 software). A correlation analysis between the expression levels of *CsNUDX1*, *CsGT1*, and *CsGT2* genes and the abundance values of geranyl *β*-primeveroside was also performed using the Pearson method in SPSS software.

## Results

### Free Geraniol in Tea Infusion and Tempo-Spatial Patterns of Geranyl Glycosides in Tea Plants

Black tea infusions made from tea cultivars “WC91,” “ZC108,” “ZN117,” and “SCZ” released high levels of geraniol into the headspace, ranging from 73.0 to 195.0 μg L^−1^ (Figures 2A,B). Qualitative analysis identified geraniol from the infusion of black teas, geranyl glucoside and primeveroside in freshly excised tea leaves by comparing the chromatography and mass spectrometry data obtained from plant samples and authentic standards (Supplementary Table S1; Supplementary Figures S1, S2). However, in this study, geraniol was not detected in the methanol extracts of plant tissues despite having over 90% recovery rate using methanol as a geraniol solvent. A quantitative analysis further revealed that the mean abundance of geranyl *β*-primeveroside in tea plants was much greater than that of geranyl *β*-glucoside, which was only 7.89% of primeveroside in first leaf of tea plants. Young leaves contained the highest levels of geranyl *β*-primeveroside, while tender roots contained the lowest levels and those in the stem and fully opened flowers were in between, indicating a tempo-spatial pattern of geranyl glycosides in tea plants (Figure 2C). Significantly less amounts of geranyl *β*-glucoside were also noted in different tea organs (Figure 2D). Moreover, the abundances of geranyl *β*-glucoside in the 10 tea cultivars were quantified, and all were at quite high levels with an average of 1.88 μg g^−1^ FW (Fresh weight); however, the abundances among these cultivars varied significantly (*p*, 0.05), and ranged from 0.84 μg g^−1^ FW to 3.07 μg g^−1^ FW (Figure 2E). Our data indicated that for many tea cultivars, geranyl *β*-glucoside accumulated in a tempo-spatial pattern and was the main glycoside of geraniol, whereas free geraniol was undetectable.

### Functional Characterization of Crucial Genes in Canonical and Noncanonical Geraniol Biosynthetic Pathways

**Figure 3:**
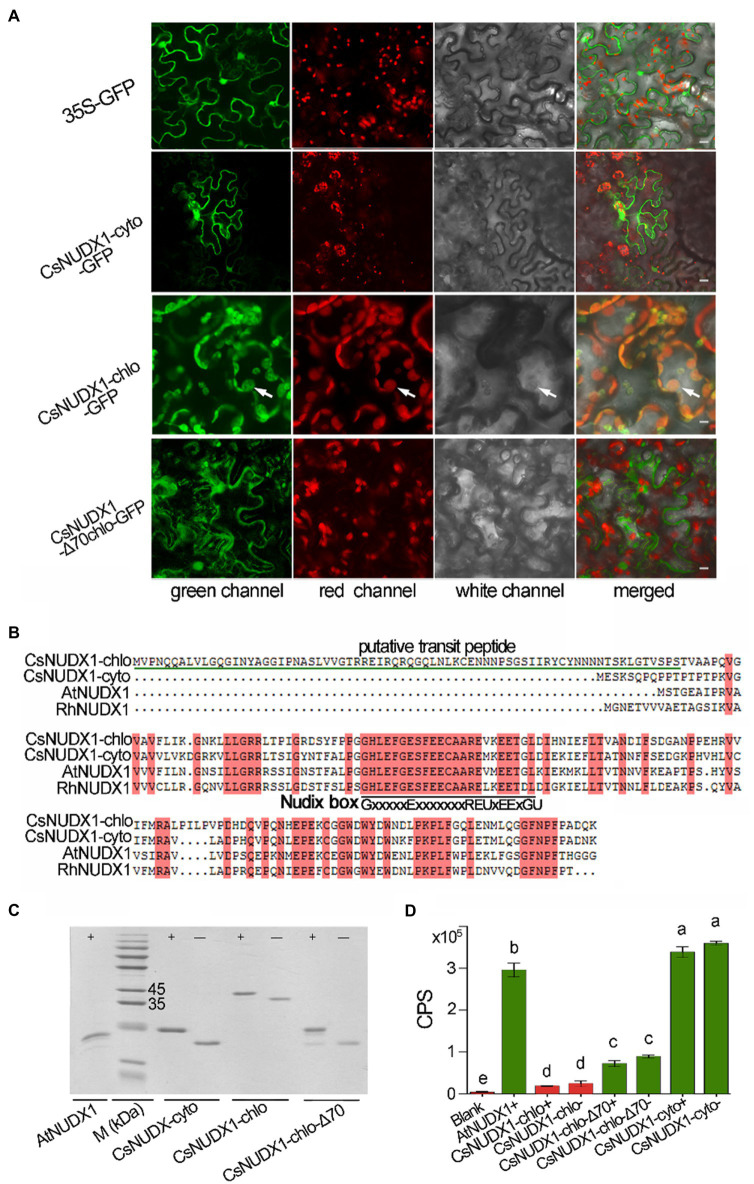
Characterization of *CsNUDX1-cyto* and *CsNUDX1-chlo*. **(A)** Subcellular localization analyses using CsNUDX1 fused with green fluorescent protein mGFP5 (GenBank U87973.1). White arrows indicate chloroplasts. All constructs were expressed in abaxial leaf surface except CsNUDX1-chlo-GFP expressed in adaxial surface. 35S-GFP, GFP gene alone driven by 35S promoter; CsNUDX1-Δ70chlo-GFP, GFP fused CsNUDX1-chlo without putative transit peptides. Scale, 10 μm. **(B)** Tea proteins aligned with the homologues of Arabidopsis (NP_177044.1) and rose (AFW17224.1). **(C)** SDS- polyacrylamide gel electrophoresis of *Escherichia coli* expressed *CsNUDX1* with/without His-tag and Arabidopsis *AtNUDX1* with His-tag as control. M (kDa), a protein marker (kilo Dalton); CsNUDX1-chlo-Δ70, CsNUDX1-chlo with deleted the putative transit peptide containing 70 amino acid residues at its N-terminus. **(D)** The levels of *in vitro* product geranyl FIGURE 3 | monophosphate (GP) produced by CsNUDIX1-cyto and CsNUDX-chlo and their recombinant variants. Columns with different letters had significant differences in the abundances of product GP (*p* < 0.05). CPS, count per second of the product ion detected. Blank, without any protein added in the reaction mixture; Protein variant plus “+” or “−” denotes protein with or without the His-tag.

Two tea homologues (TEA006189 and TEA000568) of rose RhNUDX1 (GenBank: AFW17224.1), a critical enzyme for geraniol production in rose flowers through a noncanonical pathway (Magnard et al., 2015), were retrieved from the tea genome through a BLASTP search. Both tea genes possessed the conserved Nudix box (GX5EX7REUXEEXGU, where U is a bulky hydrophobic residue and X is any residue; Liu et al., 2018b), indicating that both were members of Nudix hydrolase family. The online prediction indicated that TEA000568 contained a putative 70 amino acid chloroplast transit peptide, while TEA006189 contained no transit peptide; thus, these are presumably being targeted to the chloroplast and the cytoplasm, respectively. A *GFP* reporter gene was fused in-frame at the 3′ end of both *CsNUDX1* genes and then introduced into plant leaves along with cytoplasm control of GFP and putatively plastidial but truncated *CsNUDX1* with removed transit peptide (Figure 3A). GFP fused with putatively cytoplasmic CsNUDX1 exhibited green signals surrounding the cytoplasm, similar as the green signals of cytosolic GFP (35S-GFP). Interestingly, green signals of GFP fused with truncated CsNUDX1 without the putative transit peptide (CsNUDX1-Δ70chlo-GFP) displayed a similar cytosolic pattern of green signals in the merged view. On the contrary, GFP fused with putatively plastidial CsNUDX1 displayed its green signals with distinct subcellular location, well overlapped with red signals of chloroplasts, suggesting that it was a true chloroplastidial protein with a transit peptide containing 70 amino acid residues. Our data confirmed the predicted subcellular localizations of the two NUDX1 proteins. Thus, the two genes were thereafter named as *CsNUDX1-chlo* for *TEA000568* and *CsNUDX1-cyto* for *TEA006189*. The respective CsNUDX1-cyto and CsNUDX1-chlo proteins contain 156 and 220 amino acid residues and share high similarities to the functional homologues in Arabidopsis and rose (Figure 3B). CsNUDX1-cyto and CsNUDX1-chlo had 78 and 64% similarity with the RhNUDX1 protein, respectively. For the *in vitro* functional assay, *CsNUDX1-cyto* and *CsNUDX1-chlo* were expressed in *E. coli* and corresponding recombinant proteins with or without an N-terminally linked His-tag were obtained (Figure 3C). The Arabidopsis AtNUDX1 protein with a His-tag was also obtained as positive control. Results showed that CsNUDX1-cyto possessed a stronger GPP to GP hydrolysis activity than the Arabidopsis AtNUDX1 (*p* < 0.05; Figure 3D). Nevertheless, the hydrolysis activity of CsNUDX1-chlo with/without the His-tag was barely detectable (Figure 3D). CsNUDX1-chlo-Δ70 (with the putative transit peptide removed) exhibited an increased activity compared to the full length CsNUDX1-chlo, but its activity was still weak compared to CsNUDX1-cyto (Figure 3D).

We also made further effort to identify tea *GES* genes. *ObGES*, together with other seven functional geraniol synthase genes, were applied as query sequences to search for homologous candidate *GES* genes in the tea genome. The highest similarities were found for *CSA008212* (49% with the *Cinnamomum tenuipilum* homologue) and *TEA014987* (44% with that of *Catharanthus roseus*; Supplementary Table S2). The two candidate genes were cloned, sequenced, and transiently expressed in *N. benthamiana*, which has proved an effective approach for examination of GES activities *in planta* (Dong et al., 2013). However, compared to the buffer control, no significantly increased amounts of geraniol or its primeveroside were detected in the transgenic leaves (Supplementary Figure S3), suggesting that neither of the two possessed detectable catalytic activities for production of geraniol or its primeveroside.

### Transgenic Study on the Function of *CsNUDX1-cyto* and *CsNUDX1-chlo in Planta*

**Figure 4:**
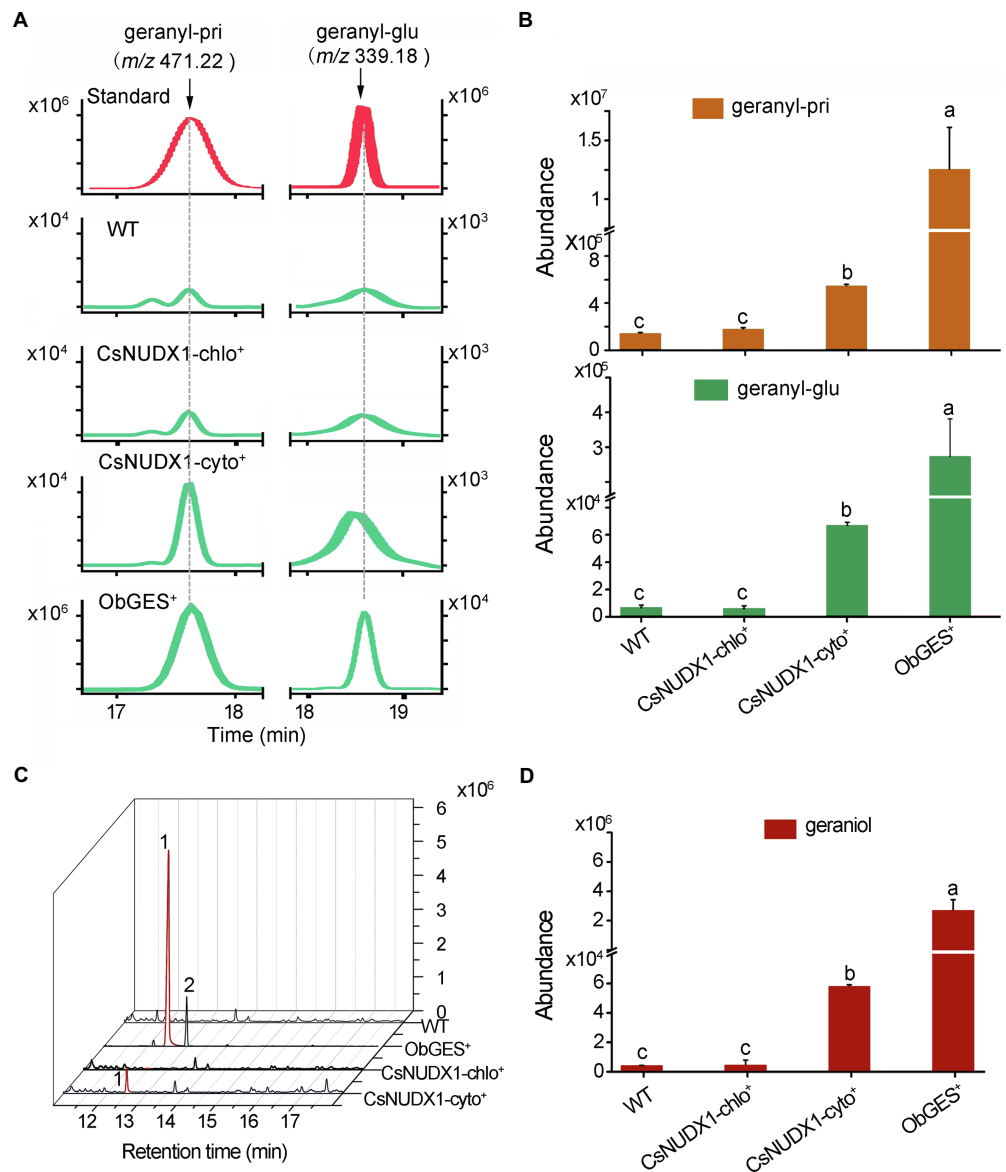
Functional characterization of the two tea *CsNUDX1s*. **(A)** Liquid chromatographs of geranyl glucoside, primeveroside found in the transgenic *Nicotiana benthamiana* leaves transiently overexpressing *CsNUDX1-cyto*, *CsNUDX1-chlo*, *ObGES* respectively, compared to non-transgenic wild-type (WT). The compounds were identified by comparing their corresponding unique ions (*m/z*) and retention times to those of the authentic standards. **(B)** Quantitative analysis of geranyl glucoside and primeveroside in different transgenic leaves. Columns with the same letters had no significant difference (*p* > 0.05). **(C)** Gas chromatograph of geraniol headspace-SPME collected from different transgenic and non-transgenic leaves after hydrolysis of glycosides with exogenous addition of glucosidase. Peaks 1 and 2, represent geraniol and citral, respectively; Geranyl-pri, geranyl *β*-primeveroside; geranyl-glu, geranyl *β*-glucoside; *ObGES^+^*, *CsNUDX1-cyto^+^*, and *CsNUDX1-chlo^+^*, the transgenics overexpressing corresponding genes; WT, non-transgenic wild-type. **(D)** Quantitative column presentation of geraniol abundances with the data obtained from **(C)**.

For further functional investigation of the two *CsNUDX1* genes in plants, *CsNUDX1-cyto* and *CsNUDX1-chlo* were each transiently expressed under control of the CaMV 35S promoter in *N. benthamiana* (*CsNUDX1-cyto^+^* or *CsNUDX1-chlo^+^*), while *ObGES* was also expressed in the same transgenic system (*ObGES^+^*) as a positive control and non-transgenic wild type (WT) as negative control. Geranyl *β*-primeveroside and geranyl *β*-glucoside were identified in the transgenic and non-transgenic leaves of *N. benthamiana* (Figure 4A). Quantitative analysis indicated that both transgenic leaves *CsNUDX1-cyto^+^* and *ObGES^+^* contained significantly higher levels of geranyl *β*-primeveroside and geranyl *β*-glucoside than WT, whereas there were no differences in the levels of two geranyl glycosides between *CsNUDX1-chlo^+^* and wildtype (Figure 4B). Furthermore, compared to the wild type and *CsNUDX1-chlo^+^* samples, significantly higher levels of released geraniol were detected from both *CsNUDX1-cyto^+^* and *ObGES^+^* leaf samples following glycoside hydrolysis with exogenous glucosidase (Figures 4C,D). Moreover, increased levels of geraniol derivatives citral and neral, as well as some other volatiles were also detected from both *CsNUDX1-cyto^+^* and *ObGES^+^* leaf samples, but not from *CsNUDX1-chlo^+^* samples. Without glycoside hydrolysis, free geraniol (as a fragment ion with *m/z* 137.133 in positive mode) was undetectable in any tested plant tissue extracts, even in *ObGES^+^* extracts in which the chemical product of *ObGES* has been demonstrated to be geraniol (Iijima et al., 2004). The precursor ion of GP (*m/z* 233.093), which is the *in vitro* product of CsNUDX1 found in this study, was not detected either. These results revealed that expression of *CsNUDX1-cyto* in transgenic leaves of *N. benthamiana* significantly increased levels of geranyl primeveroside, whereas expression of *CsNUDX1-chlo* did not lead to an increase. After CsNUDX1-cyto mediates the first step, production of geranyl primeveroside most likely results from chemical conversion *via* other endogenous enzymes.

### Functional Characterization of *CsNUDX1-cyto* in Tea Plants

**Figure 5:**
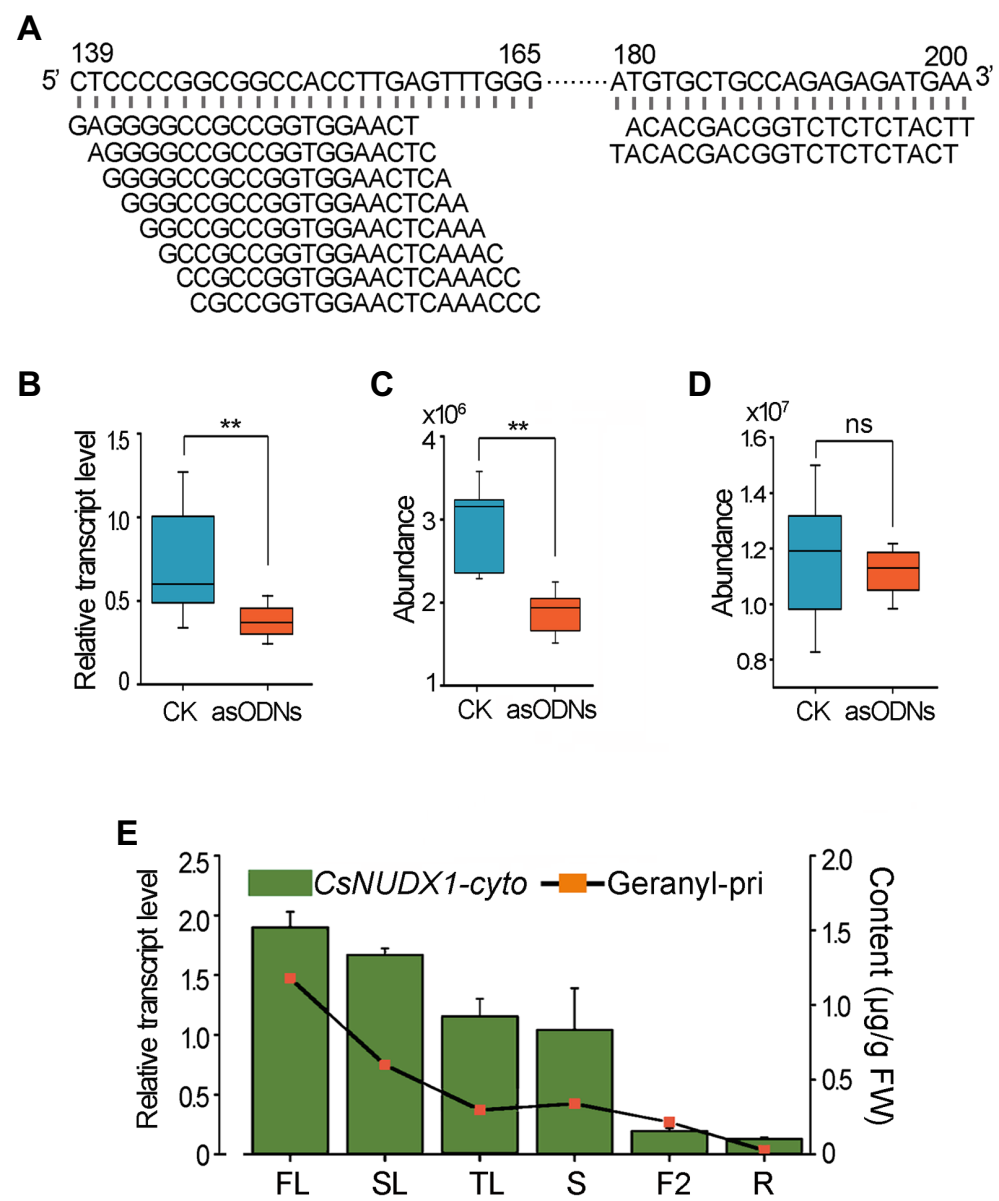
Functional characterization of *CsNUDX1-cyto* in tea plants using AsODNs approach. **(A)** Top 10 asODNs retrieved using Soligo software (Ding and Lawrence, 2003) for *CsNUDX1-cyto* suppression experiments. **(B)** A significant reduction (^**^p < 0.01) in the transcript level of *CsNUDX1-cyto* after asODNs treatment. **(C)** A significant reduction (^**^p < 0.01) in the abundance of geranyl *β*-primeveroside in the treated tea tender shoots (*p* < 0.01). **(D)** No significant change (ns) in the abundance of linalyl *β*-primeveroside in treated tea shoots compared to control (*p* > 0.05). **(E)** Highly correlated tempo-spatial transcript levels of *CsNUDX1-cyto* and the abundances of geranyl *β*-primeveroside in tea plants (correlation coefficient of 0.864). FL, SL, and TL, first, second, and third leaves; S, tender shoot stem; F2, flowers at stage 2, slightly opened; R, tender roots. *CsNUDX1-cyto*, Transcript levels of *CsNUDX1-cyto* in different organs of “SCZ” plants; Geranyl-pri, Abundances of geranyl primeveroside in different organs of “SCZ” plants.

Since tea plants are recalcitrant to *Agrobacterium*-mediated genetic transformation and *CsNUDX1-chlo* had a minor effect on geranyl *β*-primeveroside accumulation *in planta*, only *CsNUDX1-cyto* was functionally validated in tea plants using an improved gene suppression method with gene-specific asODNs. A mixture of 10 asODNs, all specifically complementary to the fragments (139–165 bp or 180–200 bp) near the 5′ end of *CsNUDX1-cyto*, was used to treat excised tender shoots of “SCZ” (Figure 5A). Quantitative real time PCR (qPCR) results showed that a 24 h treatment with asODN-CsNUDX1-cyto resulted in a significant transcript reduction (*p* < 0.01) of the target *CsNUDX1-cyto* compared to treating with the random nonsense oligo (CK; Figure 5B). Abundance of geranyl *β*-primeveroside in the treated tea leaves was significantly reduced (*p* < 0.01; Figure 5C). Notably, the abundance of linalyl *β*-primeveroside was unchanged (*p* > 0.05; Figure 5D), indicating these AsODNs specifically suppressed the target gene. Our data demonstrated that CsNUDX1-cyto functions as a key catalyst for production of geraniol and its primeveroside in tea shoots, as revealed in the transgenic tobacco plants.

In addition, a tempo-spatial transcriptional pattern of *CsNUDX1-cyto* in leaves, stem, flowers and roots in “SCZ’ plants was noted (Figure 5E). The highest transcript level was found in buds, followed by those in the other green tissues (young and mature leaves, and stems), whereas the lowest levels were in non-green tissues: fully opened flowers and tender roots. Interestingly, a similar pattern of differential geranyl primeveroside abundances were found for the above organs of “SCZ” (Figures 2C, 5E). The amounts of geranyl-primeveroside and transcript levels of *CsNUDX1-cyto* in the tested organs of “SCZ” were highly correlated in a tempo-spatial manner, as indicated by the Pearson correlation coefficient of 0.864.

## Discussion

Geraniol is a potent odorant of many tea products and is present as glycosidic conjugates in tea plants. In this study, we found that the levels of geraniol released from tea infusion are well above its perception threshold of 75 ppb (Burdock, 2001), and the finding that geranyl *β*-primeveroside was the main geranyl glycoside present in tea leaves agreed with previous findings (Wang et al., 2000). Our combined evidence from *in vitro* enzymatic assays, gene manipulation as well as gene chemical product analysis further revealed that geranyl *β*-primeveroside was enhanced due to the action of CsNUDX1-cyto, and the downstream steps likely occurred through endogenous chemical conversion by other enzymes, starting from GP-derived geraniol to geranyl *β*-primeveroside.

In tea plants, two tea homologous variants of *RhNUDX1*, a critical gene in the noncanonical pathway for geraniol biosynthesis in rose (Magnard et al., 2015), were identified in this study. Our *in vitro* assays revealed that cytosolic CsNUDX1-cyto was capable of hydrolyzing GPP to GP, while chloroplastidial CsNUDX1-chlo exhibited limited GPP hydrolysis activity even after removal of its putative transit peptide (70 amino acid residues), which is common to many other nucleus-encoded chloroplast proteins in plants (Archer and Keegstra, 1990). Protein sequence alignment further revealed that CsNUDX1-cyto shared the same amino acid residues at positions L38 and P129 as the Arabidopsis AtNUDX1 (NP_177044.1). However, CsNUDX1-chlo possessed different amino acid residues at these positions; the L38P and P129Q substitutions in CsNUDX1-chlo were most likely the reason for the significant reduction in activity of CsNUDX1-chlo, as mutations of these two positions in the Arabidopsis AtNUDIX1 almost completely abolishes the activity (Liu et al., 2018b). Thus, it may be possible that the significant difference in hydrolytic activity between the two tea NUDX1 proteins results from their protein sequences. However, a more recent finding indicated that a plastidial Nudix1 from *Tanacetum cinerariifolium* has high specificity for chrysanthemyl pyrophosphate, rather than GPP (Li et al., 2020); thus, tea CsNUDX1-chlo may be able to hydrolyze a different substrate other than GPP.

In this study, transgenic leaves of *N. benthamiana* expressing *CsNUDX1-cyto* produced significantly more geranyl primeveroside than the non-transgenic wild type, whereas that expressing *CsNUDX1-chlo* had the same geranyl primeveroside levels as wild type. This indicates that the distinct *in vitro* hydrolytic activities of the two CsNUDX1s would be the consequence of their sequence-dependent functions rather than their varying subcellular locations in plant cells, as there are GPP pools in both the cytoplasm and chloroplasts (Dong et al., 2013; Ueoka et al., 2020). Moreover, transgenic data also suggested that the chemical conversion of the *CsNUDX1-cyto* product GP occurred in the transgenic plants. Compared to non-transgenic wild type, expression of *ObGES* in transgenic *N. benthamiana* leaves, whose chemical product is geraniol (Iijima et al., 2004), resulted in a significant increase in geranyl *β*-primeveroside instead of geraniol. This indicated that the geraniol produced from *ObGES* overexpression was effectively converted into geranyl *β*-primeveroside in the transgenic *ObGES^+^ N. benthamiana* leaves due to endogenous glycosylation by the host plant. Expression of *CsNUDX1-cyto* also enhanced geranyl *β*-primeveroside production in transgenic *N. benthamiana* leaves, suggesting that the same chemical conversion from geraniol to the primeveroside would also occur in *CsNUDX1-cyto* expressing transgenics. Meanwhile, GP was verified *in vitro* as the direct chemical product of CsNUDX1-cyto, and was most likely converted to geraniol due to the action of unknown endogenous phosphatases as suggested in a rose hybrid (Magnard et al., 2015). Interestingly, *CsNUDX1-cyto* enhancement of geranyl *β*-primeveroside was comparatively less than that produced in *ObGES* expressed transgenics (*p* < 0.05), and this was probably because *CsNUDX1-cyto* is less effective than *ObGES*. Our data also showed that expression of *CsNUDX1-chlo*, which was unable to convert GPP to GP *in vitro*, did not result in an increase in either geraniol or geranyl primeveroside in transgenic *CsNUDX1-chlo^+^*; this confirms in an opposite way that chemical conversion from geraniol to its primeveroside did not occur when GP production was not enhanced. Furthermore, after addition of exogeneous glycosidase to release glycosidically bound volatiles, there was increased emission of geraniol and its derivative citral in both transgenics *CsNUDX1-cyto^+^* and *ObGES^+^* relative to the non-transgenic control. This not only confirms the occurrence of the endogenous glycosylation of geraniol in both transgenics, but also suggests that in addition to chemical conversion from GP to geraniol and to geranyl primeveroside, other geraniol chemical modifications such as geraniol oxidation would also be occurring. All our transgenic study results clearly demonstrated that *CsNUDX1-cyto* is involved in geranyl primeveroside formation through the intermediate metabolites GP, geraniol and subsequent geraniol glycosylation, although the endogenous enzymes catalyzing these reactions have yet to be identified.

Furthermore, functional validation of *CsNUDX1-cyto* in tea plants was conducted in this study using an improved gene suppression method with gene-specific asODNs (Liu et al., 2018a) to overcome the recalcitrance of tea plants to *Agrobacterium*-mediated genetic transformation. Our data confirmed that geranyl *β*-primeveroside is the chemical product of *CsNUDX1-cyto* in tea plants, as it was in transgenics *CsNUDX1-cyto^+^* or *ObGES^+^*. Additionally, similar tempo-spatial patterns of *CsNUDX1-cyto* expression and geranyl primeveroside abundance in the plants of the tea cultivar “SCZ” were found with high levels in young and green tissues and low levels in fully opened flowers and roots. The gene expression levels and geranyl *β*-primeveroside abundances among different tissues were well correlated. These data suggested that in tea plants, *CsNUDX1-cyto* could be involved in geraniol and its primeveroside biosynthesis in a noncanonical pathway.

More recently, geraniol has been reported to be sequentially glycosylated into glucoside and primeveroside due to the high substrate selectivity of the endogenous glycosyl transferases GT1 (to geraniol) and GT2 (to geranyl glucoside), respectively (Ohgami et al., 2015). We quantified transcript levels of *CsGT1,* and *CsGT2* and abundances of geranyl primeveroside in young and mature leaves as well as in tender roots in the plants of tea cultivars “Zijuan” and “WC91,” and found that their transcript levels were well correlated with the primeveroside abundance in “Zijuan” (correlation coefficients were 0.976 and 0.997 for *CsGT1* and *CsGT2*, respectively) and in “WC91” (0.866 and 0.930 for *CsGT1* and *CsGT2*, respectively); thus, our data support the observation of Ohgami et al. (2015). In the genome of *N. benthamiana*,^4^ multiple homologous genes of *CsGT1* and *CsGT2* were found. Four genes *NbS00016902g0104.1*, *NbS00057453g0005.1*, *NbS00044548g0005.1*, and *NbS00 057453g0006.1* are homologues of *GT1* and *NbS00 006161g0001.1* and *NbS00007621g0001.1* are those of *GT2*. All shared high identities in protein sequences (>60%). These are likely involved in biosynthesis of geranyl primeveroside in *N. benthamiana.* Further investigations through gene manipulation are required to validate the roles of the two sugar moiety transferase genes in geranyl *β*-primeveroside production in tea plants.

So far, no functional geraniol synthase (*GES*) genes acting in the canonical pathway in tea plants have been reported. In the current work, two of the tea *GES* candidates found through homology searches did not result in any increase in geraniol and its glycoside abundances in the transient transgenic study. Functional characterization of all annotated monoterpene synthase genes (Zhou et al., 2020) is underway to search for tea geraniol synthase, since the chemical function of a terpene synthase cannot be predicted because subtle changes in protein sequence could result in distinct product (Bohlmann et al., 1999). However, no *GES* gene has been reported in *Arabidopsis* thus far (Chen et al., 2011), though *Arabidopsis* possesses detectable geranyl glycoside (Sugimoto et al., 2015); thus, one cannot exclude the possibility that the canonical terpene pathway for geraniol biosynthesis *via* GES may not exist in some plants.

## Data Availability Statement

The original contributions presented in the study are included in the article/**Supplementary Material**, further inquiries can be directed to the corresponding authors.

## Author Contributions

SW, T-JL, GL, and HZ conceived the project. HZ and SW did most of the experiments. T-JL and JP did the prokaryotic expression and *in vitro* assays. H-FX and T-JL verified authentic standards using nuclear magnetic resonance spectroscopy. ZZ completed geranyl glucoside synthesis. LS completed the manuscript draft. T-JL and SW finalized the manuscript. All authors contributed to the article and approved the submitted version.

## Funding

The research described here was funded by the National Natural Science Foundation of China (Grant Number 31370687) to SW and (Grant Number 32002096) to HZ.

## Conflict of Interest

The authors declare that the research was conducted in the absence of any commercial or financial relationships that could be construed as a potential conflict of interest.

## Publisher’s Note

All claims expressed in this article are solely those of the authors and do not necessarily represent those of their affiliated organizations, or those of the publisher, the editors and the reviewers. Any product that may be evaluated in this article, or claim that may be made by its manufacturer, is not guaranteed or endorsed by the publisher.

## Footnotes

^1^ www.chemhui.cn

^2^ www.cbs.dtu.dk/services/ChloroP/

^3^ www.ebi.ac.uk/Tools/msa/clustalw2/

^4^ solgenomics.net/organism/Nicotiana_benthamiana/genome
