# Supplementary material for: Cytosolic Nudix Hydrolase 1 Is Involved in Geranyl β-Primeveroside Production in Tea
Source: Front Plant Sci. 2022 May 11;13:833682. doi: 10.3389/fpls.2022.833682 (PMC9131077; doi:10.3389/fpls.2022.833682)
Supplement: Supplementary file 1 [file Data_Sheet_1.docx]

Supporting files for

Cytosolic *Nudix hydrolase 1* is involved in geranyl *β*-primeveroside production in tea

Hanchen Zhou^1,2#^, Shijie Wang^1#^, Hao-Fen Xie^1^, Guofeng Liu^1,3^, Lubobi Ferdinand Shamala^1^, Jingyi Pang^1^, Zhengzhu Zhang^1^, Tie-Jun Ling^1*^, Shu Wei^1*^

^1^State Key Laboratory of Tea Plant Biology and Utilization, Anhui Agricultural University, Hefei 230036, China

^2^ Tea Research Institute, Anhui Academy of Agricultural Sciences, Huangshan, China

^3^ Henan Provincial Key Laboratory of Tea Plant Biology, Xinyang Normal University, Xinyang, 464000, Henan, China

*** Correspondence:**SW, [weishu@ahau.edu.cn](mailto:weishu@ahau.edu.cn) and TJL, [lingtj@ahau.edu.cn](mailto:lingtj@ahau.edu.cn)

^#^ equal contributors to this work.

Supporting Tables

**Table S1.** Structural confirmation of synthesized geranyl *β*-primeveroside using nuclear magnetic resonance spectroscopy (in CD_3_OD, *δ* in ppm, *J* in Hz).

| position | *δ*_C_ (sample) | *δ*_C_ (literature) ^[1,2,3]^ | *δ*_H_ (sample) | *δ*_H_ (literatures) ^[1,2,3]^ |
| --- | --- | --- | --- | --- |
| 1 | 66.6 | 66.6 | 4.22 (1H, dd, 7.6, 11.8) | 4.22 (1H, dd, 7.3, 11.7) |
|  |  |  | 4.35 (1H, m) | 4.34 (1H, dd, 6.6, 11.7) |
| 2 | 121.5 | 121.6 | 5.38 (1H, br t, 6.5) | 5.37 (1H, br t, 5.1) |
| 3 | 141.9 | 142.0 | − | − |
| 4 | 40.7 | 40.7 | 2.05 (2H, m) | 2.05 (2H, m) |
| 5 | 27.4 | 27.5 | 2.13 (2H, m) | 2.11 (2H, m) |
| 6 | 125.1 | 125.1 | 5.11 (1H, br t, 6.8) | 5.11 (1H, br t, 5.1) |
| 7 | 132.5 | 132.6 | − | − |
| 8 | 25.9 | 25.9 | 1.68 (3H, s) | 1.61, 1.67, 1.69 (each 3H, s) |
| 9 | 17.8 | 17.8 | 1.61 (3H, s) |  |
| 10 | 16.6 | 16.6 | 1.69 (3H, s) |  |
| 1' | 102.9 | 103.0 | 4.29 (1H, d, 7.9) | 4.28 (1H, d, 8.1) |
| 2' | 75.0 | 75.2 | 3.18−3.23 (1H, m)^a^ | 3.15−3.25 (1H, m)^c^ |
| 3' | 78.0 | 78.2 | 3.30−3.36 (1H, m)^b^ | 3.29−3.35 (1H, m)^d^ |
| 4' | 71.5 | 71.7 | 3.30−3.36 (1H, m)^b^ | 3.29−3.35 (1H, m)^d^ |
| 5' | 77.0 | 77.0 | 3.41 (1H, m) | 3.40 (1H, m) |
| 6' | 69.8 | 69.9 | 3.75 (1H, dd, 6.0, 11.4) | 3.74 (1H, dd, 5.9, 11.7) |
|  |  |  | 4.09 (1H, dd, 1.8, 11.4) | 4.08 (1H, dd, 2.2, 11.7) |
| 1'' | 105.5 | 105.6 | 4.32 (1H, d, 7.6) | 4.31 (1H, d, 7.3) |
| 2'' | 74.9 | 75.0 | 3.18−3.23 (1H, m)^a^ | 3.15−3.25 (1H, m)^c^ |
| 3'' | 77.7 | 77.9 | 3.30−3.36 (1H, m)^b^ | 3.29−3.35 (1H, m)^d^ |
| 4'' | 71.2 | 71.3 | 3.49 (1H, m) | 3.48 (1H, m) |
| 5'' | 66.9 | 67.0 | 3.18−3.23 (1H, m)^a^ | 3.15−3.25 (1H, m)^c^ |
|  |  |  | 3.86 (1H, dd, 5.3, 11.5) | 3.85 (1H, dd, 5.2, 11.0) |
| ^a b c d^, Signals were overlapped. | | | | |

Geranyl *β*-primeveroside


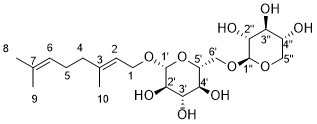


[1] Guo W, Sakata K, Watanabe N, et al. Geranyl 6-*O*-*β*-D-xylopyranosyl-*β*-D-glucopyranoside isolated as an aroma precursor from tea leaves for oolong tea. Phytochemistry, 1993, 33 (6): 1373-1375.

[2] Tian J, Zhang H J, Sun H D, et al. Monoterpenoid glycosides from *Ligustrum robustum*. Phytochemistry, 1998, 48(6): 1013-1018.

[3] Yu Y, Gao H, Dai Y, et al. Monoterpenoids from the fruit of *Gardenia jasminoides*. Helvetica Chimica Acta, 2010, 93(4): 763-771.

**Table S2.** Search for potential tea geraniol synthase genes homologous to identified functional genes from different plant species (Identities >40%).

| **Species** | | **Protein ID** | | **Length(aa)** | | **CSA008212.1 (600aa)** | | | | | **TEA014987.1(607aa)** | | |
| --- | --- | --- | --- | --- | --- | --- | --- | --- | --- | --- | --- | --- | --- |
|  |  |  |  |  |  | **Query coverage** | | **Identities** | **Query coverage** | | | | **Identities** |
| *Perilla frutescens* | ABB30218.1 | | 603 | | 89% | | 255/547 (47%) | | | 87% | | 212/547 (39%) | |
| *Ocimum basilicum* | AAR11765.1 | | 567 | | 84% | | 189/508(37%) | | | 78% | | 198/482 (41%) | |
| *Vitis vinifera* | ADR74217.1 | | 591 | | 80% | | 211/485(44%) | | | 82% | | 214/506 (42%) | |
| *Cinnamomum tenuipilum* | CAD29734.2 | | 603 | | 89% | | 268/544(49%) | | | 88% | | 232/543 (43%) | |
| *Phyla dulcis* | ADK62524.1 | | 584 | | 81% | | 193/493(39%) | | | 87% | | 205/489 (42%) | |
| *Catharanthus roseus* | AFD64744.1 | | 589 | | 77% | | 194/467(42%) | | | 79% | | 213/486 (44%) | |
| *Perilla citriodora* | AAY88965.1 | | 603 | | 89% | | 257/547(47%) | | | 87% | | 212/547 (39%) | |

**Table S3.** Primers used in this study.

| **Primer Name** | | | **Sequence** | | **Usage** | |  |
| --- | --- | --- | --- | --- | --- | --- | --- |
| CsNUDX1-chlo-F-XbaI | | tctagaatggtgcccaatcagcaagctt | | Full length gene cloning | | | |
| CsNUDX1-chlo-R-SacI | | gagctctcatttttgatcagctggaaaggga | |  |  |  |  |
| CsNUDX1-cyto-BamHI-F | | ggatccatggaaagcaagtctcagc | |  | | | |
| CsNUDX1-cyto-SacI-R | | gagctctcatttattatcagctggg | |  | | | |
| NUDX1-chlo-F | | gatatcatggtgcccaatcagcaagctt | | For prokaryotic analysis | | | |
| CsNUDX1-chlo-R | | ggatccgagattaggcaaaggcaaggcca | |  | | | |
| CsNUDX1-cyto-F | | ggatccatggaaagcaagtctcagc | |  | | | |
| CsNUDX1-cyto-R | | gagctctcatttattatcagctggg | |  | | | |
| CsNUDX1-chlo-GFP-F | | ttccagctgatcaaaaatcgggtggaggcagcatgagtaaaggagaaga | | For subcellular localization | | | |
| CsNUDX1-chlo-Δ70-GFP-F | | tctacaatgccgtcgacggtgg | |  | | | |
| CsNUDX1-chlo-GFP-R | | tcttctcctttactcatgctgcctccacccgatttttgatcagctggaa | |  | | | |
| CsNUDX1-cyto-GFP-F | | tcccagctgataataaatcgggtggaggcagcatgagtaaaggagaaga | |  | | | |
| CsNUDX1-cyto-GFP-R | | tcttctcctttactcatgctgcctccacccgatttattatcagctgga | |  | | | |
| GFP-R | | gagctcttatttgtatagttcatccatgccatgt | | | | | |
| CsNUDX1-chlo-Q-F | gccaccttgagtttggggagag | | | For qPCR analysis | |  |  |
| CsNUDX1-chlo-Q-R | cacgatgttctggtggatttgctc | | |  | |  |  |
| CsNUDX1-cyto-Q-F | tcttggctgatcctcatcaagtacc | | |  | |  |  |
| CsNUDX1-cyto-Q-R | atcagctgggaaaggattaaaacca | | |  | |  |  |

Supporting figures


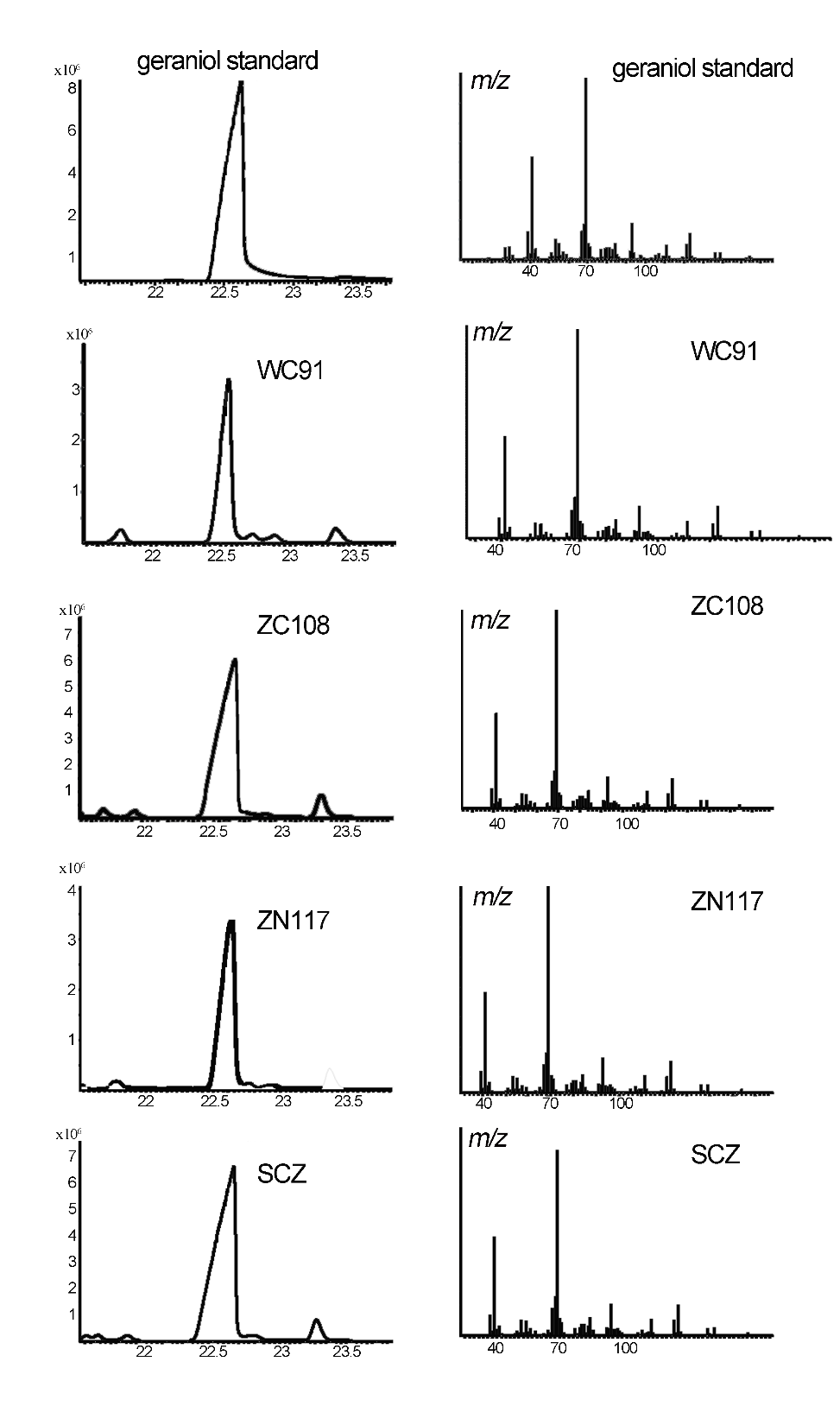


**Fig. S1**. Identification of geraniol in tea infusions using GC-MS and authentic standards of geraniol.


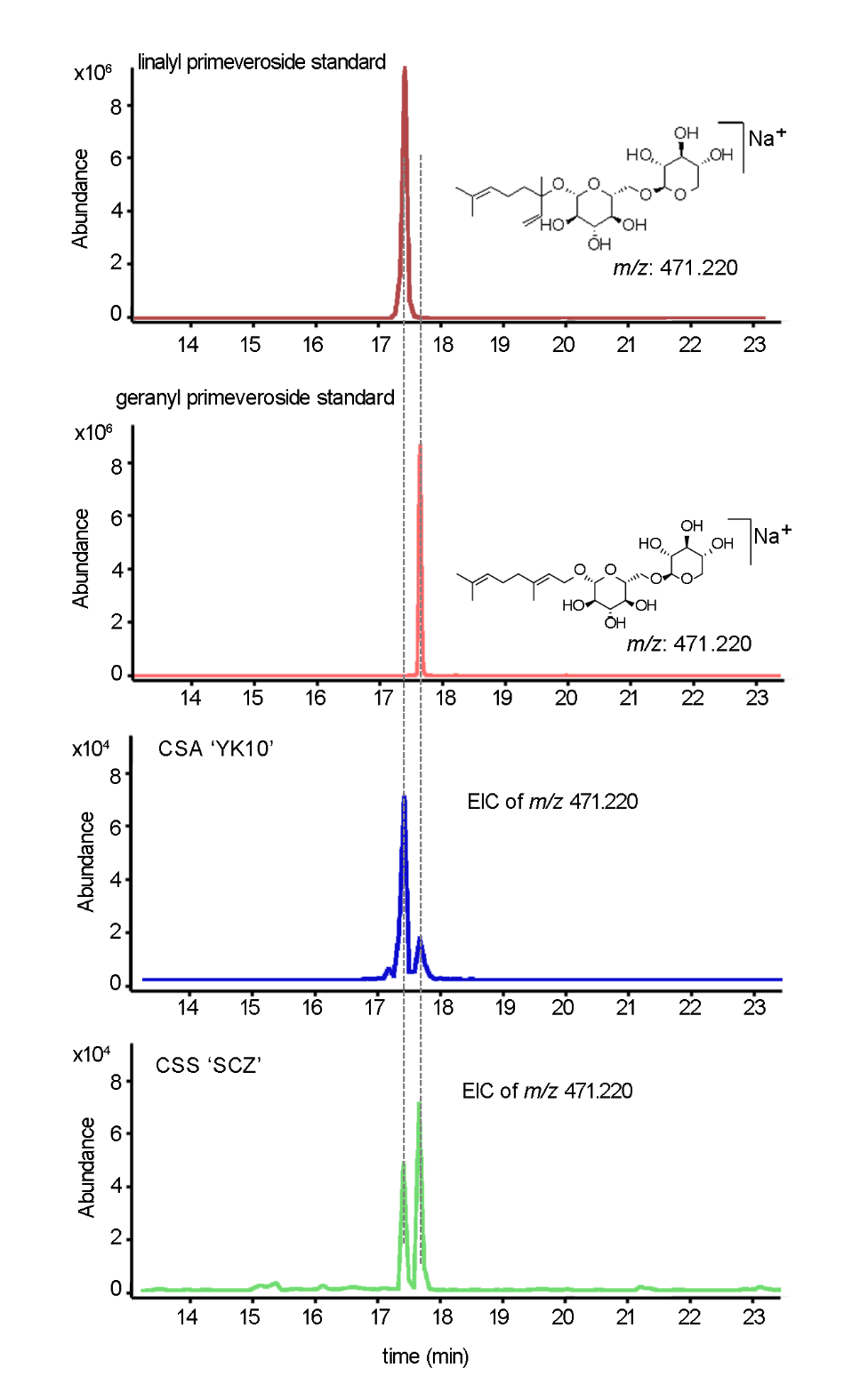
**Fig. S2.** Identification of geranyl- and linalyl primeveroside in tea plants using the ultra-high-performance liquid chromatography-quadrupole time-of-flight mass spectrometry (UHPLC/Q-TOF-MS) technique and authentic standards of the two di-glycosides. Geranyl- and linalyl primeveroside have the same precursor ion of *m/z* 471.220, but their retention times are 17.78 min and 17.63 min, respectively.


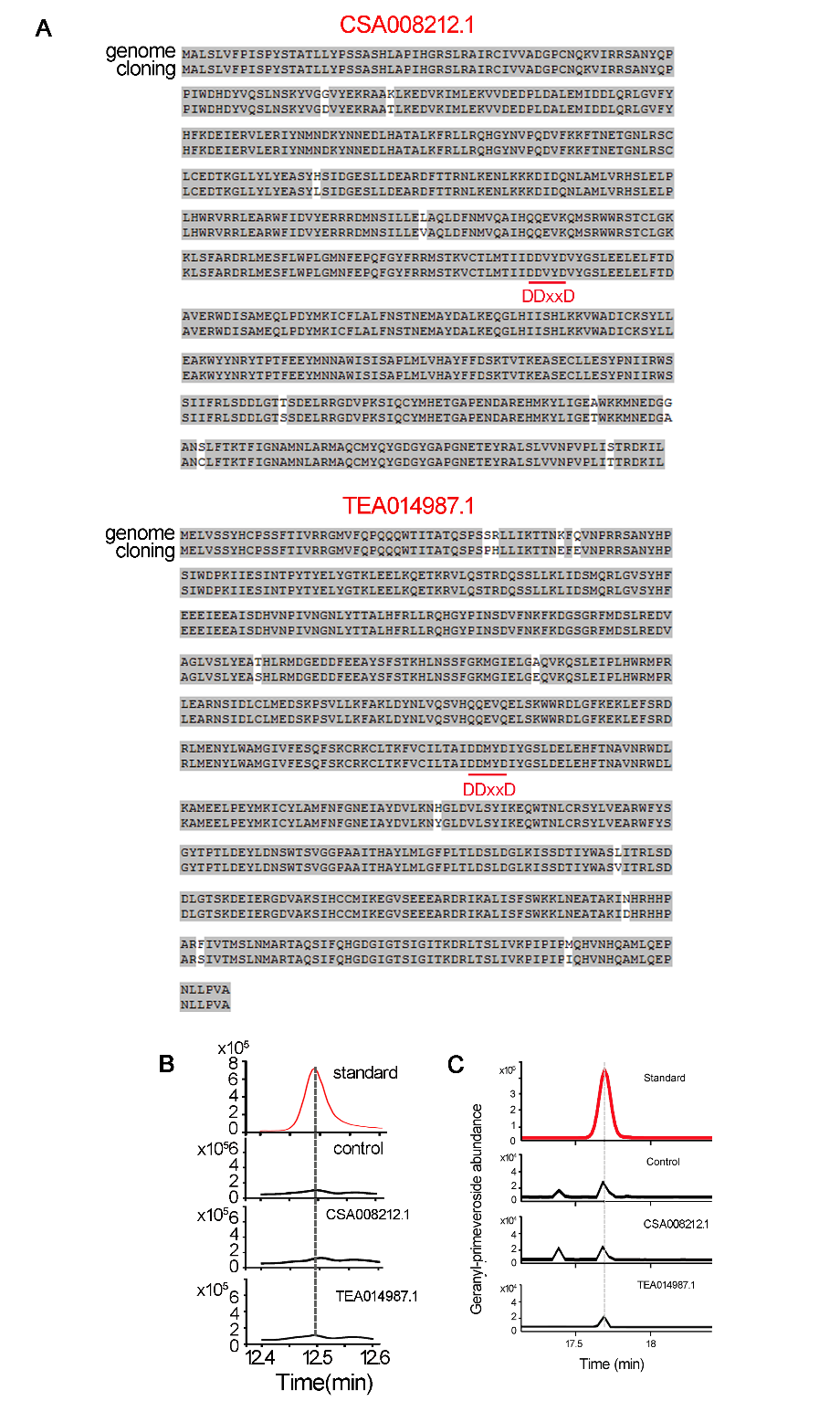


**Fig. S3.** Search for tea homologues via BLASTP using functional geraniol synthase gene as query. **A** Sequencing results of the two tea genes found by BLASTP search using ObGES as query. **B** GC-MS analysis of geraniol in the transgenic *N. benthamiana* leaves after glucosidase hydrolysis. **C** UPLC-MS/MS analysis of geranyl-primeveroside in the transgenic *N. benthamiana* overexpressing the two tea genes (*CSA008212.1* and *TEA014987.1*) leaves compared with wild-type control.
